# Supplementary figures and images for: Collection and Storage of Human Plasma for Measurement of Oxylipins
Source: Metabolites. 2021 Feb 26;11(3):137. doi: 10.3390/metabo11030137 (PMC7996814; doi:10.3390/metabo11030137)

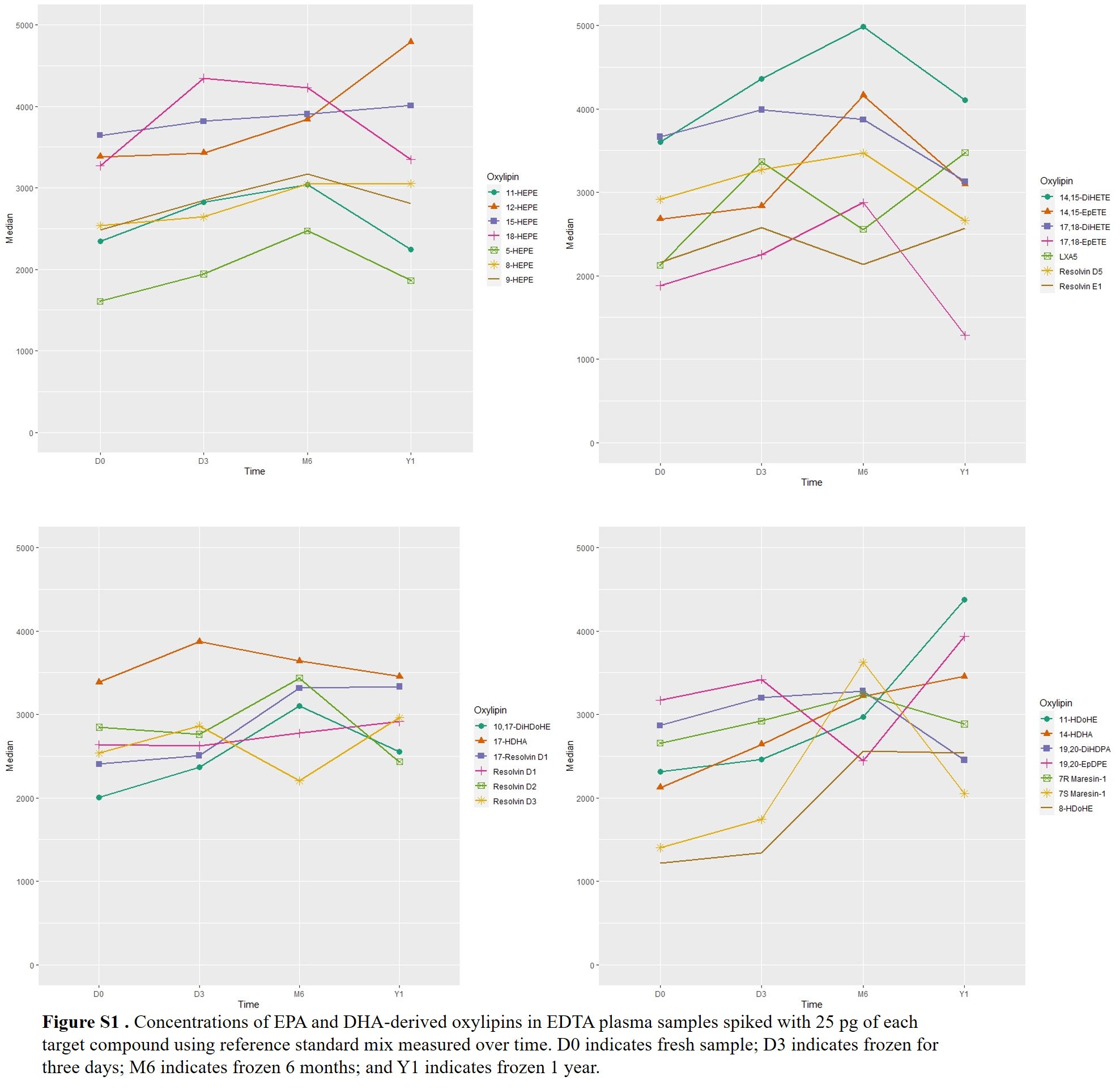

Supplement: Supplementary file 1 [file metabolites-11-00137-s001.zip › Figures final/S1_figure.jpg]

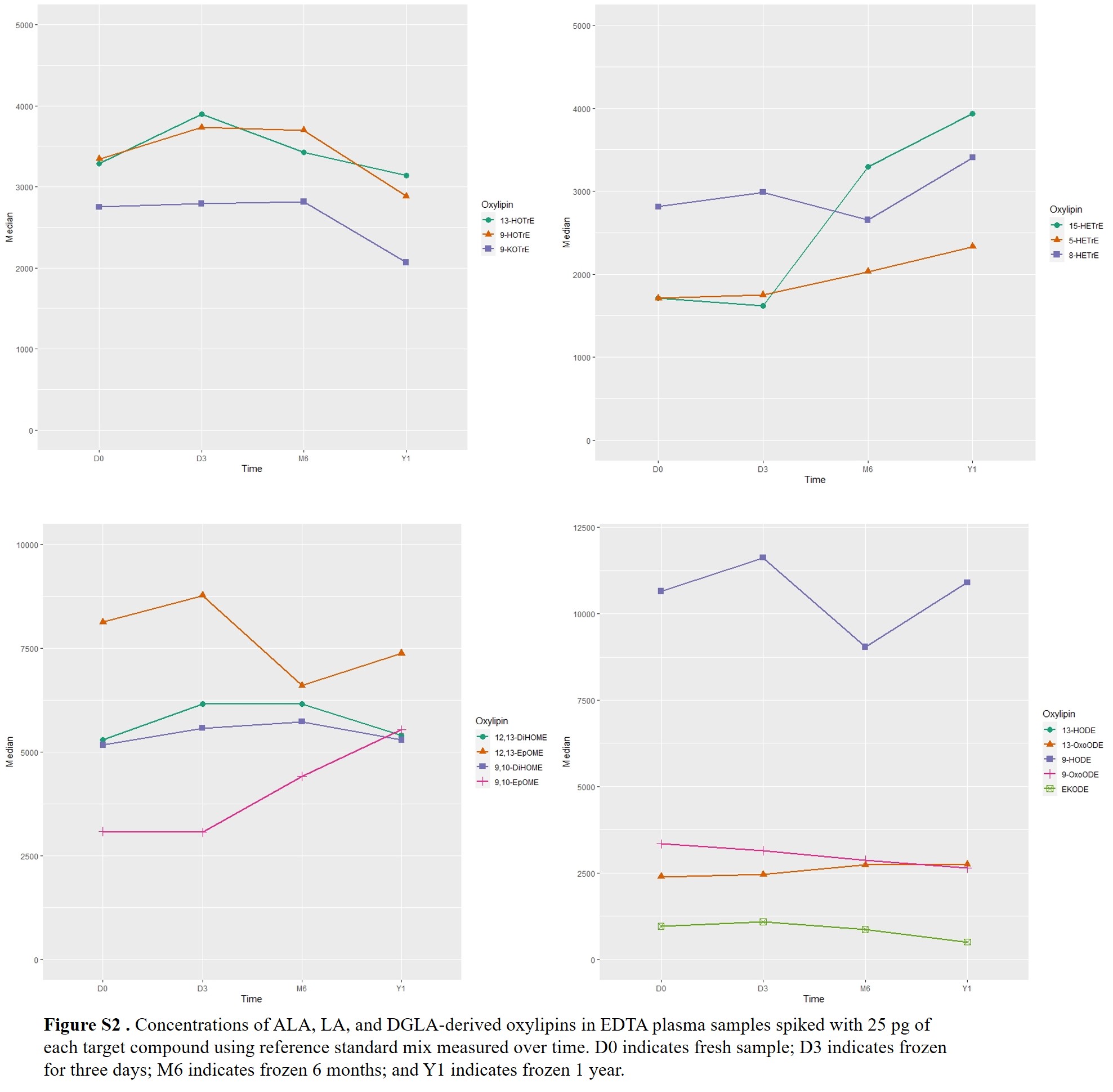

Supplement: Supplementary file 1 [file metabolites-11-00137-s001.zip › Figures final/S2_figure.jpg]

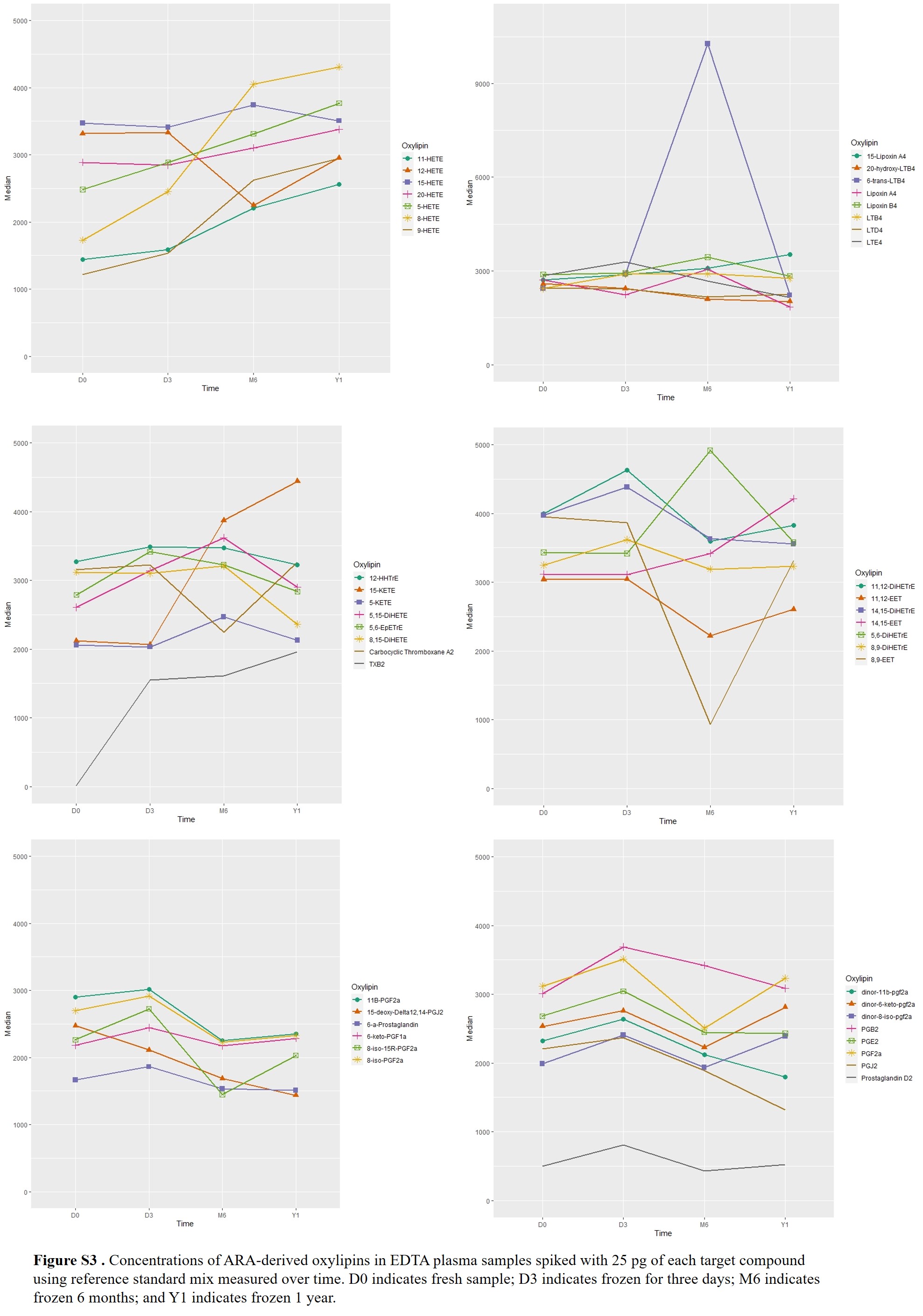

Supplement: Supplementary file 1 [file metabolites-11-00137-s001.zip › Figures final/S3_figure.jpg]
